# Is NO the Answer? The Nitric Oxide Pathway Can Support Bone Morphogenetic Protein 2 Mediated Signaling

**DOI:** 10.3390/cells8101273

**Published:** 2019-10-18

**Authors:** Christopher Differ, Franka Klatte-Schulz, Nicole Bormann, Susann Minkwitz, Petra Knaus, Britt Wildemann

**Affiliations:** 1 Julius Wolff Institute, Charité – Universitätsmedizin Berlin, Corporate Member of Freie Universität Berlin, Humboldt-Universität zu Berlin, and Berlin Institute of Health, 13353 Berlin, Germany; christopher.differ@outlook.com (C.D.); franka.klatte@charite.de (F.K.-S.); Nicole.bormann@charite.de (N.B.); susann.minkwitz@charite.de (S.M.); 2 Berlin-Brandenburg School for Regenerative Therapies, Charité – Universitätsmedizin Berlin, 13353 Berlin, Germany; 3 Berlin-Brandenburg Center for Regenerative Therapies, Charité – Universitätsmedizin Berlin, 13353 Berlin, Germany; petra.knaus@fu-berlin.de; 4 Institute of Chemistry and Biochemistry – Free University, 14195 Berlin, Germany; 5 Experimentelle Unfallchirurgie – Universitätsklinikum Jena, 07747 Jena, Germany

**Keywords:** C2C12, BMP2, nitric oxide, signaling, crosstalk

## Abstract

The growth factor bone morphogenetic protein 2 (BMP2) plays an important role in bone development and repair. Despite the positive effects of BMP2 in fracture healing, its use is associated with negative side effects and poor cost effectiveness, partly due to the large amounts of BMP2 applied. Therefore, reduction of BMP2 amounts while maintaining efficacy is of clinical importance. As nitric oxide (NO) signaling plays a role in bone fracture healing and an association with the BMP2 pathway has been indicated, this study aimed to investigate the relationship of BMP2 and NO pathways and whether NO can enhance BMP2-induced signaling and osteogenic abilities in vitro. To achieve this, the stable BMP reporter cell line C2C12BRELuc was used to quantify BMP signaling, and alkaline phosphatase (ALP) activity and gene expression were used to quantify osteogenic potency. C2C12BRELuc cells were treated with recombinant BMP2 in combination with NO donors and substrate (Deta NONOate, SNAP & L-Arginine), NOS inhibitor (LNAME), soluble guanylyl cyclase (sGC) inhibitor (LY83583) and activator (YC-1), BMP type-I receptor inhibitor (LDN-193189), or protein kinase A (PKA) inhibitor (H89). It was found that the NOS enzyme, direct NO application, and sGC enhanced BMP2 signaling and improved BMP2 induced osteogenic activity. The application of a PKA inhibitor demonstrated that BMP2 signaling is enhanced by the NO pathway via PKA, underlining the capability of BMP2 in activating the NO pathway. Collectively, this study proves the ability of the NO pathway to enhance BMP2 signaling.

## 1. Introduction

Bone morphogenetic proteins (BMPs) provide approximately 20 members to the transforming growth factor beta (TGFβ) superfamily. BMPs are archetypal growth factors capable of inducing diverse cellular responses such as proliferation, survival, apoptosis, differentiation, and migration, and play an important role in the development and homeostasis of bone [1]. BMPs act not only in the skeleton, but also have roles in other tissue types, for example, in tendons [2], nerve synapses [3], and endothelial cells [4].

BMPs have become clinically relevant [5], particularly in the orthopedic discipline [6,7,8], where BMP2 is licensed for the treatment of open tibia fractures [9]. Clinical trials highlight the ability of BMP2 to improve health outcomes of patients with open tibia shaft fracture by significantly reducing healing times, complications, infections, further operations, and hardware failures compared to the standard care group [10,11]. A clinical trial of rhBMP2 in the treatment of open tibia fractures did not find a clear improvement in fracture healing. However, rhBMP2 reduced the failure rates of surgical interventions, therefore reducing the need for further clinical interventions [8].

With current clinical applications of BMP2 being 1000 times greater than physiological levels, it is not surprising that negative effects, such as poor bone structure and excess inflammation, have become apparent [12,13,14]. Clinical trials have indicated issues with the effectiveness of BMP2 and negative effects, such as increased signs of edema and deep venous thrombosis [15] or prolonged inflammatory response (wound drainage) [16]. The Orthopedic Research Society Forum [14] stated that the excessive clinical doses of rhBMP2 (compared to normal physiological amounts) could be the underlining issue in negative outcomes and that there is a need to refine and reduce rhBMP2 application in the clinic. One possibility is to optimize BMP2 treatment through the enhancement of BMP-signaling by modulating this pathway.

BMP2 binds dimeric BMP receptor type 1 (BMPR1a, BMPR1b) and type 2 (BMPR2, ActR2A, ActR2B), thus forming hetero-tetrameric receptor complex [17,18]. The affinities between BMP2 and the various receptors differ, generating complexity and redundancy in BMP signaling [19].

The long C-terminal cytoplasmic tail of the type 2 receptor BMPR2 contains valuable binding sites for adapter proteins [20,21,22]. This extra binding allows for interactions with additional proteins that lead to modulations of signaling processes such as CD117, tribbles 3 (Trb-3), protein phosphatase 2 (PP2), protein kinase A (PKA), and cGMP dependent kinase I (cGKI) [23,24,25,26,27].

The NO pathway effector kinase, cGKI, was shown to play a role at many points of BMP signaling. cGKI associates with and phosphorylates the BMPR2 tail-domain [28]. Once the receptor is activated by ligand binding, cGKI leaves the receptor to associate with and enhance the action of SMAD transcription factors [26,29]. In pulmonary arterial endothelial cells, PKA was described as a crucial link between the NO- and BMP-pathway [27]. Endothelial cells treated with BMP2/4 activate PKA via BMPR2 which, in turn, mediates phosphorylation of the endothelial NO synthase (eNOS) [27]. Here, we asked whether there is an association between the NO- and BMP2-pathways in the context of BMP2-mediated osteo-inductive activity. This might have important implications for improvement of BMP2-based therapeutics.

The NO pathway has been associated with osteo-inductive activities. The application of a NOS inhibitor (LNAME) to rats following bone fracture led to reduction in bone healing, which was recovered by direct NO donation [30]. The application of nitrates (e.g., nitroglycerin) has been shown to reduce osteopenia and osteoporosis in animal models [31,32,33]. In clinical trials, nitrate-treated female osteoporotic patients showed an increase in osteogenic markers and bone density [34,35,36]. This association between the NO and BMP2 pathway could prove valuable in improving BMP2-mediated osteogenic activities. For this purpose, the BMP2 sensitivity [37] and osteogenic features [38,39,40] of the mouse C2C12 myoblast cell line were exploited to investigate whether the NO pathway combines and enhances the effects of the BMP2 pathway, and if the BMP2 pathway is capable of modulating the NO pathway.

## 2. Materials and Methods

### 2.1. Cell Culture

The C2C12BRELuc reporter cell line [41] was generated by the Inman group and used in this study. In this cell line, the luciferase (Luc) reporter is under the control of the BMP response elements (BRE) of the *Id1* gene. C2C12BRELuc were cultivated in culture medium (Dulbecco’s modified Eagle’s medium (DMEM) supplemented with 10% heat-inactivated fetal bovine serum (FBS) and antibiotics (100 U/mL penicillin and 100 μg/mL streptomycin, all from Biochrom AG, Berlin, Germany). C2C12BRELuc cells were passaged twice per week in a 175 or 225 cm^2^ cell culture flask. The cultivation media was supplemented with the antibiotic G418 (0.7 mg/mL) to select for the BREluc-positive C2C12BRELuc cells in accordance with previous studies [41].

C2C1BRELuc cells were sub-cultivated at a ratio of 2.5–5 × 10^4^ cells per mL of culture medium. DMEM, antibiotics, FBS, and trypsin (Biochrom AG, Berlin, Germany) were used in cell cultivation and passaging. To ensure the viability and to calculate cell number, the metabolic activity of cells was calculated through Presto Blue^®^ assay (Invitrogen by Life Technologies Co., Carlsbad, CA, USA). BMP2 was purchased from Osteogenetics (Würzburg, Germany). H89 was purchased from AbCam (Cambridge, UK). Deta NONOate, SNAP, YC-1, LY83583, LDN-193189, and IBMX were purchased from Cayman Chemical (Ann Arbor, MI, USA).

### 2.2. BMP Reporter Assay

The experimental setup utilized 0.1% FCS, as it allows C2C12 cells to respond rapidly to BMP2. Cells were seeded at a density of 2.5 × 10^4^ cells per well in culture media into 48-well cell culture dishes overnight. The medium was exchanged to Dulbecco's Modified Eagle Medium (DMEM) without l-Arginine (GIBCO^®^ by Life Technologies GmbH, Darmstadt, Germany) supplemented with 0.1% FCS and 1% Pen/Strep for starvation for three hours. Subsequently, medium was changed to the appropriate stimulation medium for the stated period of time (see Table 1 for experimental setup). The medium was removed after the stimulation period and the cells were lysed in 100 μL lysis buffer (1:5 Cell Lysis Reagent diluted in ddH_2_O; Promega, Mannheim, Germany), frozen at −20 °C, and subsequently thawed at room temperature on a shaker. The cell lysate (20 µL) was then transferred to a 96-well plate and 50 µL of Luciferase buffer (Promega, Mannheim, Germany) was added by automatic injection using a luminometer (Mithras LB-940, Berthold Technologies, Bad Wildbad, Germany). Quantification was determined as relative light units (RLU). For protein normalization, the protein content of lysates was determined with the Pierce™ Coomassie assay (Thermo Fisher Scientific, Darmstadt, Germany) and Bovine Serum Album (BSA) as standard. The assay was carried out following manufactures instruction. Standards were made in PBS (or appropriate experimental buffer) and samples were diluted 1:2 in PBS and measured in triplicates.

### 2.3. Alkaline Phosphatase Activity (ALP) Assay

C2C12BRELuc cells were seeded at a density of 2 × 10^4^ per well in a 24-well plate and incubated in DMEM High Glucose (Biochrom AG, Berlin, Germany) supplemented with 10% FCS and 1% Pen/Strep. Prior to starvation, the cell number was quantified using Presto Blue^®^ Assay. After starvation with 1% FCS, 1% Pen/Strep in DMEM/Ham’s F-12 (Biochrom AG, Berlin, Germany) for three hours, cells were stimulated with the same medium for three days (Table 1). Following stimulation, the medium was aspirated and cells were washed with pre-warmed PBS. ALP activity was analyzed by the addition of 1 mL of ALP buffer (50 mM glycine, 100 mM TRIZMA base, 1 mM MgCl_2_, pH 10.5) containing 1.3 mg pNPP (Sigma-Aldrich, Hamburg, Germany) per well. After incubation for one hour at 37 °C, 180 µL medium was transferred to a clear 96-well plate in triplicates and the absorbance was quantified at 405 nm using a spectrophotometer.

### 2.4. Gene Expression Analysis

For expression analysis of *inhibitor of differentiation (Id), 1, 2,* and *3*, 5 × 10^5^ cells were seeded to six-well cell culture dishes in culture medium. The next day, cells were starved for three hours in starving medium. The cells were then stimulated for six hours (Table 1).

Osteogenic differentiation was determined by expression analysis of *osteoprotegerin* (*OPG*), *collagen type I alpha 1 chain* (*Col1a1*), and *runt-related transcription factor 2* (*Runx2*). A total of 1.2 × 10^5^ cells was cultivated in culture medium overnight and starved with DMEM without Arginine (supplemented with 1% FCS and 1% antibiotics) for three hours. Stimulation was done for six days with a complete medium change after three days (Table 1).

RNA isolation was carried out using the NucleoSpin^®^ RNA kit (Macherey-Nagel, Düren, Germany) following the instructions. The RNA was dissolved in 30 µL RNAase Free H_2_O and RNA concentration was measured using a spectrophotometer.

Typically, 500 ng RNA was translated into cDNA using qScript cDNA SuperMix (Quantabio, Beverly, MA, USA) according to the manufacturer’s protocol. The mRNA expression of the genes of interest was quantified using quantitative real-time polymerase chain reaction (qRT-PCR). The reaction mixture was composed of 25–50 ng cDNA with 10 µM primers (see Table 2, TIB-MOLBIOL Syntheselabor GmbH, Berlin, German), nuclease-free H_2_O, and PerfeCTa SYBR^®^ Green SuperMix (Quantabio, Beverly, MA, USA). Next, 15 µL of the reaction mixture was transferred to a white 96-well qRT-PCR plate and LightCycler 480 System (Roche, Mannheim, Germany) was used. The amplification protocol included 40 cycles with an annealing temperature at 60 °C, followed by a melting curve protocol until 95 °C with a ramp rate of 0.11 °C/sec. The efficiency was tested for all primers and the ΔCt method with efficiency correction was used to calculate the relative gene expression to the housekeeping gene *hypoxanthine-guanine phosphoribosyl-transferase (HPRT)*. *HPRT* was chosen as a housekeeping gene as it was most constant over the different stimulation conditions compared to other housekeeping genes such as *Actin* (NM_009608.3), *Eukaryotic Translation Elongation Factor 1 alpha 2* (NM_010106), *Ribosomal protein L13* (NM_016738.5), *18S rRNA* (NR_003278.3), and *Glycerinaldehyd-3-phosphat-Dehydrogenase* (NM_001289726.1). For exemplary melting curves of qRT-PCR see Figure S1. 

### 2.5. NO Quantification

NO quantification protocol was modified from Hinchee-Rodriguez et al. 2013 [42]. C2C12BRELuc cells were seeded at a density of 6 × 10^4^ cells per well of a 24-well plate with DMEM High Glucose 10% FCS and 1% Pen/Strep and incubated overnight. Following starvation, cells were loaded for one hour with 5 µM DAF-2 (Biomol GmbH, Hamburg, Germany) in serum-free PBS. Subsequently, cells were stimulated for two hours in DMEM High Glucose supplemented with 1% FCS and 1% Pen/Strep. Following stimulation, cells were washed twice with warm PBS and fluorescence was quantified using a spectrophotometer at 485 nm excitation and 538 nm emission. The direct NO donor DetaNONOate was employed as a positive NO control.

### 2.6. cGMP ELISA

C2C12BRELuc cells were seeded in six-well plates with 6 × 10^5^ cells per well in culture medium overnight. Three hours of starvation and one hour of stimulation were carried out in DMEM without Arginine with 1% FCS. The inhibitor of cGMP degradation IBMX (500 µM) was added during starvation and stimulation to all samples. Post-stimulation, cells were washed with cold PBS, aspirated, and 500 µL 0.1 M HCL was added. After 10 min incubation at room temperature, the plate was stored at −20 °C. Subsequent thawing was followed by scrapping of cells. The lysates were then aliquoted into protein LoBind tubes (Eppendorf, Hamburg, Germany) and centrifuged at 600× *g* for 10 min at 4 °C.

The cGMP ELISA (NewEast Biosciences, King of Prussia, PA, USA) assay was carried out following the standard protocol from the manufacturer and absorption was measured at 450 nm for HRP and 570 nm reference using a spectrophotometer. The normalization of samples was carried out using Coomassie assay.

### 2.7. SMAD-1 ELISA

C2C12BRELuc cells were seeded with 5 × 10^4^ cells per well in a 24-well plate and incubated in culture medium overnight. The next day, cells were starved in DMEM without Arginine, supplemented with 0.1% FCS and 1% antibiotics for three hours. One hour before starving ended and during stimulation, the BMP type-I receptor inhibitor LDN was supplemented to the inhibitor group. Stimulation was carried out with the same medium supplemented with BMP2 and Arginine (Table 1) for 30 min.

Phosphorylated and total SMAD1 were measured with the SMAD1 InstantOne ELISA™ kit, Invitrogen by Life Technologies Co., Carlsbad, CA, USA. Cells were incubated with Cell Lysis Mix (1:5 Cell Lysis Reagent diluted in ddH_2_O, provided in the kit) for 10 min at room temperature and lysates were transferred to protein LoBind tubes. Samples were stored on ice and ELISA was directly carried out after sample taking following manufacturers instruction. Absorption was measured with a spectrophotometer at 450 nm with reference at 570 nm. Phosphorylated SMAD1 was normalized to total SMAD1. 

### 2.8. Statistics

All experiments were done in triplicate and repeated at least once (except for the PCR). The statistical analyses were performed using IBM^®^ SPSS^®^ Statistics version 22. All graphs were visualized as boxplots. The mild outliers are marked with a circle and extreme outliers are marked with a star. The nonparametric Kruskal-Wallis test was used to determine if there were statistically significant differences between all groups. In cases of significances during Kruskal-Wallis tests, a comparison between two groups was made by Mann-Whitney *U* test. The significances are given in the figures and are assigned if *p* < 0.05 (*), *p* < 0.01 (**), or *p* < 0.001 (***).

## 3. Results

C2C12 is a myoblast precursor cell line, which is responsive to BMPs [37] and possess osteogenic features [38,39,40]. The stable transfection of C2C12 cells with a luciferase (Luc) reporter composed of SMAD-binding BMP response elements (BRE) has provided a valuable tool in the quantification of BMP activity [41]. In order to investigate the possible NO-BMP crosstalk, Arginine-free DMEM was used to have better control over the NO pathway.

### 3.1. BMP2-Mediated Signaling and Osteogenic Activity Can be Enhanced by Arginine Via NOS

#### 3.1.1. Arginine Supports BMP2-Mediated Activity

**Figure 1:**
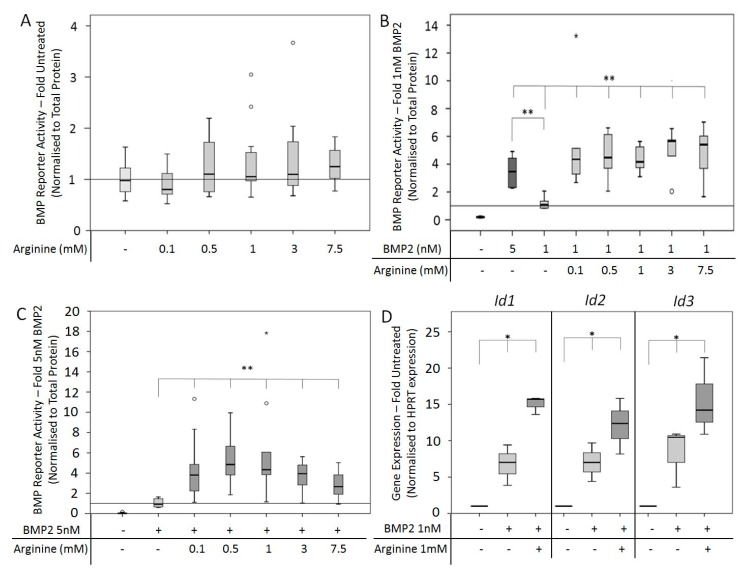
Bone morphogenetic protein (BMP) reporter activity and BMP target gene expression after stimulation with the nitric oxide synthase (NOS) substrate Arginine. C2C12BRELuc cells stimulated for 24 h in Arginine-free Dulbecco's Modified Eagle Medium (DMEM) with (**A**) 0.1 to 7.5 mM Arginine alone or in combination with (**B**) 1 nM BMP2 or (**C**) 5 nM BMP2. (**D**) C2C12BRELuc cells were stimulated for six hours in Arginine-free DMEM with 1 nM BMP2, with or without 1 mM Arginine. The gene expression of *Id1*, *Id2*, and *Id3* was investigated. Statistics: Kruskal–Wallis Test followed by Mann-Whitney *U* test. Mann-Whitney *U* test significances between samples were assigned if *p* < 0.05 (*) or *p* < 0.01 (**). Stars and circles are outlying values. Number of replications: (**A**) n = 12; (**B**) n = 6; (**C**) n = 9; (**D**) n = 3.

Arginine, a substrate of the NOS enzyme, was investigated to determine its effect on BMP2 signaling. After 24 h of stimulation, C2C12BRELuc cells showed no increase in reporter activity in response to supplementation of Arginine alone (Figure 1A). Arginine supplementation in combination with 1 nM or 5 nM BMP2 demonstrated a significant increase in BMP2-induced BMP reporter activity (Figure 1B,C). Co-stimulation of the cells with 1 nM BMP2 and Arginine was as effective as stimulation with 5 nM BMP2 alone. The expression of the immediate BMP2 response genes *Id1*, *Id2*, and *Id3* was examined following six hours of stimulation with 1 nM BMP2 with or without 1 mM Arginine (Figure 1D). The stimulation with BMP2 increased expression of the target genes as expected, which was further stimulated by the application of the NOS substrate Arginine (Figure 1D).

#### 3.1.2. Arginine Support of BMP2 Activity is Mediated by NOS

**Figure 2:**
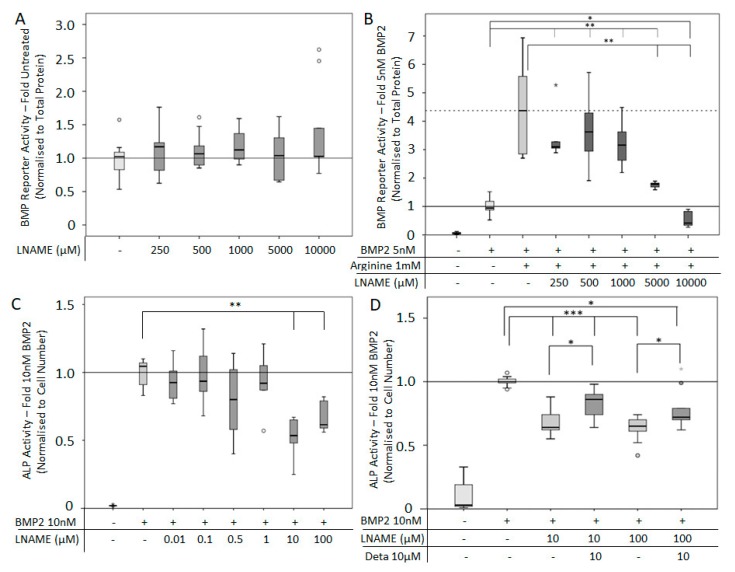
BMP reporter and alkaline phosphatase activity (ALP) activity following the inhibition of NOS enzyme by NOS inhibitor (LNAME): C2C12BRELuc cells were stimulated for 24 h with (**A**) 250-10,000 µM LNAME or (**B**) 5 nM BMP2 and 1 mM Arginine in combination with 250-10,000 µM LNAME. ALP activity was normalized to cell number of cells stimulated for 72 h with 10 nM BMP2 with (**C**) NOS inhibitor LNAME (0.01 to 100 µM) and (**D**) following recovery of 10 µM or 100 µM LNAME inhibition with the direct NO donor. Statistics: Kruskal–Wallis Test followed by Mann-Whitney *U* test. Mann-Whitney *U* test significances between samples were assigned if *p* < 0.05 (*) or *p* < 0.01 (**) or *p* < 0.001 (***). Stars and circles are outlying values. Abbreviation: Deta: Deta NONOate. Number of replications: (**A**–**D**) n = 6.

To further demonstrate that the application of Arginine supports BMP2-mediated SMAD signaling via the NO pathway, the inhibitor of the NOS enzyme (LNAME) was applied to C2C12BRELuc cells. The application of LNAME (250 µM–10000 µM; high concentration in accordance to [43]) alone had no effect on the BMP reporter activity (Figure 2A). The Arginine-enhanced BMP2 signaling was significantly reduced by LNAME (5,000 µM and 10,000 µM) (Figure 2B). ALP activity was utilized to investigate the osteoinductive effect of BMP2. For this assay, cells were cultured for three days. The application of LNAME at 10 and 100 µM reduced significantly the BMP2-mediated ALP activity (Figure 2C). Subsequently, LNAME inhibition of BMP2-mediated ALP activity was recovered by the direct application of direct NO donor Deta NONOate (Figure 2D).

### 3.2. BMP2-Mediated Signaling and Osteogenesis Can be Enhanced by Direct NO Donor

**Figure 3:**
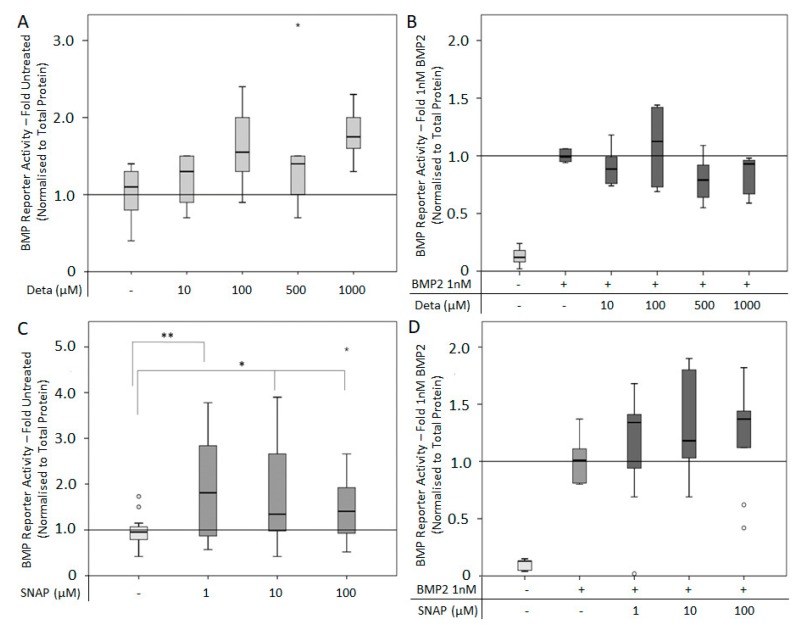
BMP reporter activity after stimulation with the NO donors Deta NONOate and SNAP for six hours: C2C12BRELuc cell were stimulated in Arginine-free DMEM with (**A**) 10–1000 µM Deta NONOate or (**B**) in combination with 1 nM BMP2, with (**C**) 1-100 µM SNAP or (**D**) SNAP in combination with 1 nM BMP2. Statistics: Kruskal–Wallis Test followed by Mann-Whitney *U* test. Mann-Whitney *U* test significances between samples were assigned if *p* < 0.05 (*) or *p* < 0.01 (**). Abbreviations: Deta = Deta NONOate. Stars and circles are outlying values. Number of replications (**A**,**B**) n = 6; (**C**) n = 18; (**D**) n = 9.

The NO donors Deta NONOate and SNAP release NO spontaneously at different rates. Deta NONOate has a half-life of 20 h, whereas SNAP has a half-life of six hours. The use of Deta NONOate (10–1000 μM) alone or in combination with 1 nM BMP2 for six hours had no significant effect on BMP reporter activity (Figure 3A,B). Application of SNAP (1–100 μM) alone significantly increased BMP reporter activity compared to untreated controls (Figure 3C), while co-application of SNAP (1–100 μM) with 1 nM BMP2 had no significant effect on reporter activity compared to BMP2 alone (Figure 3D).

**Figure 4:**
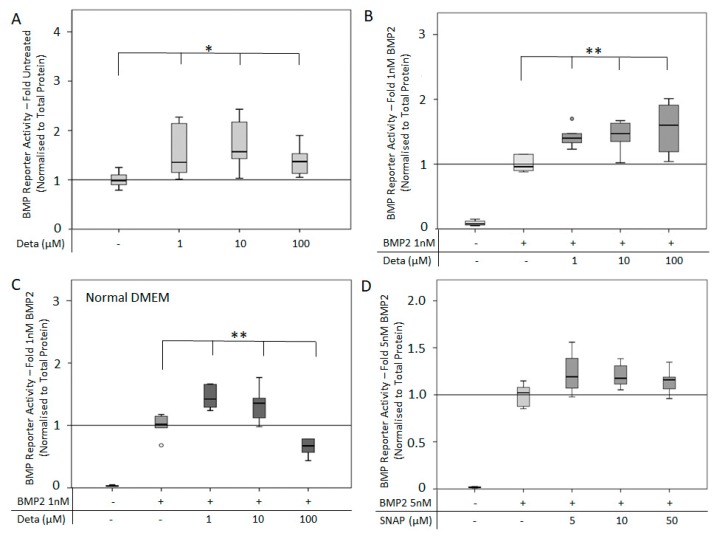
BMP reporter activity after stimulation with the NO donors Deta NONOate and SNAP for 24-h: C2C12BRELuc cells stimulated in Arginine-free DMEM with (**A**) 1–100 µM Deta NONOate, (**B**) and in combination 1 nM BMP2. (**C**) Cells stimulated in normal DMEM containing Arginine with 1 to 100 µM Deta NONOate and 1 nM BMP2. (**D**) Stimulation in Arginine-free DMEM with 5–50 µM SNAP in combination with 5 nM BMP2. Statistics: Kruskal–Wallis Test followed by Mann-Whitney *U* test. Mann-Whitney *U* test significances between samples were assigned if *p* < 0.05 (*) or *p* < 0.01 (**). Abbreviation: Deta = Deta NONOate. Circles are outlying values. Replication (**A**–**D**) n = 6.

Due to the fact that no effect was observed after six hours, the experiment was repeated with a stimulation period of 24 h. For this 24 h timepoint, Deta NONOate alone (1–100 μM) stimulated BMP reporter activity (Figure 4A). The combined use of Deta NONOate (1–100 μM) and 1 nM BMP2 significantly increased BMP reporter activity compared to BMP2 alone in a dose-dependent manner (Figure 4B). Interestingly, in Arginine-containing media, a lower dose of Deta NONOate (1 and 10 μM) with 1 nM BMP2 had an additional effect on the cells, while a high concentration of Deta NONOate (100 μM) and 1nM BMP2 was inhibitory (Figure 4C). The combination of SNAP (5–50 μM) with 1 nM BMP2 had no effect on reporter activity compared to BMP2 alone (Figure 4D).

**Figure 5:**
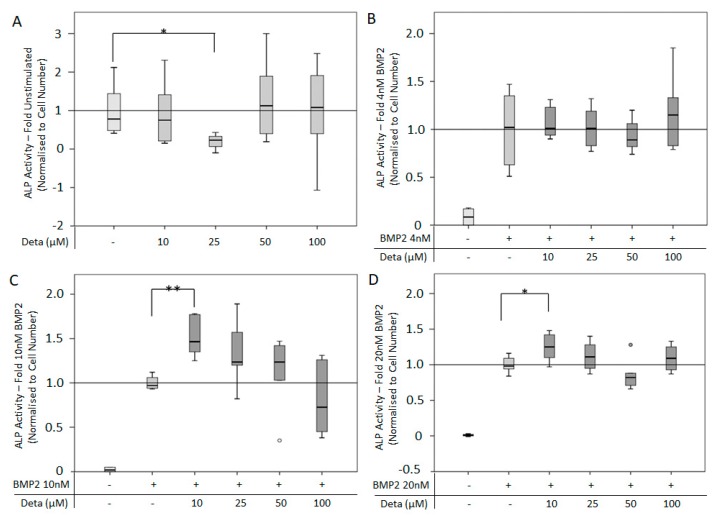
ALP activity after NO supplemented BMP2 stimulation: ALP activity normalized to cell number of C2C12BRELuc cells stimulated with direct NO donor Deta NONOate (10–100 µM) (**A**) without BMP2, (**B**) with 4 nM BMP2, (**C**) with 10 nM BMP2, or (**D**) with 20 nM BMP2. Statistics: Kruskal–Wallis Test followed by Mann-Whitney *U* test. Mann-Whitney *U* test significances between samples were assigned if *p* < 0.05 (*) or *p* < 0.01 (**). Abbreviation: Deta = Deta NONOate. Circles are outlying values. Replications (**A**,**B**) n = 6.

The ALP activity of C2C12BRELuc cells was used as an outcome measure of osteogenic differentiation. Since Arginine-free media was unable to mediate an increase of ALP activity in response to BMP2, cells were cultured in DMEM-HAMs with 1% FCS [44]. Stimulation of cells with Deta NONOate (10–100 µM) for 72 h showed no significant difference in ALP activity compared to untreated cells except for a reduction of activity by 25 µM Deta NONOate (Figure 5A). The co-stimulation of cells with different concentrations of BMP2 (4, 10, 20 nM) and Deta NONOate (10–100 µM) revealed a complex relationship depending on the concentration combinations. Only the low Deta NONOate concentration (10 µM) in combination with 10 and 20 nM BMP2 significantly stimulated ALP activity (Figure 5B–D). The stimulatory effect was more pronounced at 10 nM BMP2 than at 20 nM BMP2.

**Figure 6:**
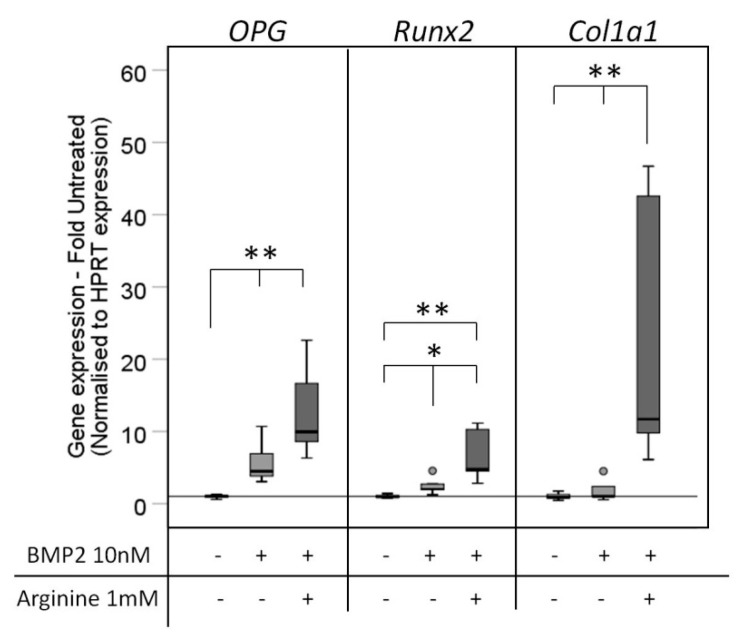
Osteogenic gene expression after stimulation with the NOS substrate Arginine. C2C12BRELuc cells were stimulated for six days in Arginine-free DMEM with 10 nM BMP2 alone or in combination with 1 mM Arginine. The gene expression of *OPG, Runx2*, and *Col1a1* was investigated by qRT-PCR. Statistics: Kruskal–Wallis Test followed by Mann-Whitney *U* test. Mann-Whitney *U* test significances between samples were assigned if *p* < 0.05 (*) or *p* < 0.01 (**). Circles are outlying values. Number of replications: n = 6.

The effect of NO donation on BMP mediated osteogenic differentiation was further tested in a six-day stimulation experiment in Arginine-free DMEM supplemented with BMP2 with and without Arginine. BMP significantly stimulated the expression of *OPG* and *Runx2* compared to the unstimulated control, while *Col1a1* expression was not affected. Further Arginine supplementation significantly enhanced the BMP-mediated osteogenic differentiation as determined by *Runx2*, *Col1a1* expression, without reaching significant differences in *OPG* expression (Figure 6).

Taken together, BMP signaling, as well as BMP-induced ALP activity and expression of osteogenic markers of C2C12 cells, can further be enhanced by a NO donor. 

### 3.3. NO Receptor, sGC, Assists in BMP2 Induced Signaling

**Figure 7:**
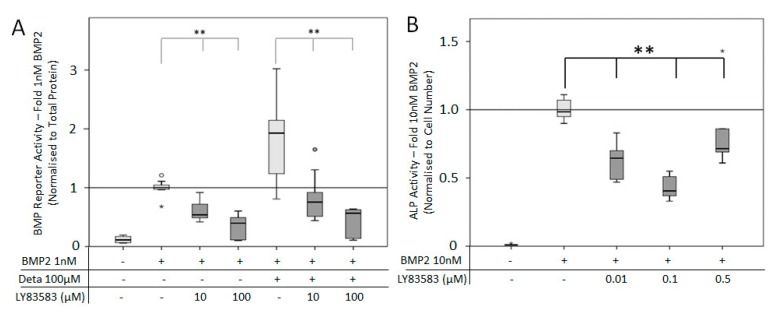
BMP reporter and ALP activity after inhibition of soluble guanylyl cyclase (sGC): C2C12BRELuc cell line was stimulated for 24 h in DMEM without Arginine (**A**) with 10 or 100 µM LY83583 in the presence of 1 nM BMP2 with and without 100 µM Deta NONOate. (**B**) ALP activity normalized to cell number after stimulation for 72 h in DMEM/Hams with 10 nM BMP2 and sGC inhibitor LY83583 (0.01 to 0.5 µM). Statistics: Kruskal–Wallis Test followed by Mann-Whitney *U* test. Mann-Whitney *U* test significances between samples were assigned if *p* < 0.05 (*) or *p* < 0.01 (**). Abbreviation: Deta = Deta NONOate. Stars and circles are outlying values. Replications (**A**) n = 9, (**B**) n = 6.

The NO pathway relies on the NO receptor, soluble guanylyl cyclase (sGC), as part of the signal transduction pathway. Therefore, understanding the effects of inhibition (LY83583, Figure 7) and NO-independent activation (YC-1, Figure 7) of this receptor is important to demonstrate the effect of the NO pathway on supporting BMP2 signaling activity. C2C12BRELuc cells were treated with LY83583 and 1 nM BMP2, with and without 100 μM Deta NONOate (Figure 7A). Both concentrations of LY83583 (10 and 100 μM) significantly reduced BMP2 signaling without a further effect of direct NO application (Figure 7A). The addition of LY83583 (0.01–0.5 μM) reduced significant the BMP induced ALP activity (Figure 7B). At the three-day period required for ALP activity, high concentrations of LY83538 (10–100 μM) resulted in a decrease in cell number, either due to the longer time or change in media type.

**Figure 8:**
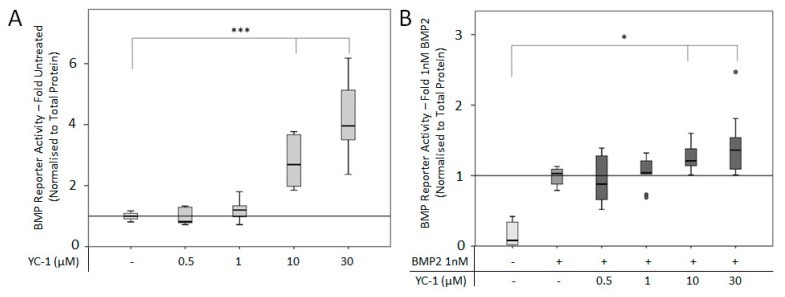
BMP reporter activity following NO independent activation of sGC: C2C12BRELuc reporter cell line stimulated for 24 h in DMEM without Arginine with (**A**) 0.5-30 µM YC-1 and (**B**) in combination with 1 nM BMP2. Statistics: Kruskal–Wallis Test followed by Mann-Whitney *U* test. Mann-Whitney *U* test significances between samples were assigned if *p* < 0.05 (*) or *p* < 0.001 (***). Circles are outlying values. Replications (**A**, **B**) n = 9.

To further investigate the direct connection between sGC and BMP2-mediated signaling, the NO-independent activator YC-1 was applied to C2C12BRELuc cells in combination with 1 nM BMP2 (Figure 8). The addition of 10 and 30 µM YC-1 alone significantly increased BMP reporter activity (Figure 8A). A similar trend was seen when additional BMP2 was added to the cultures (Figure 8B).

### 3.4. SMAD Signaling after BMP and NO Stimulation

**Figure 9:**
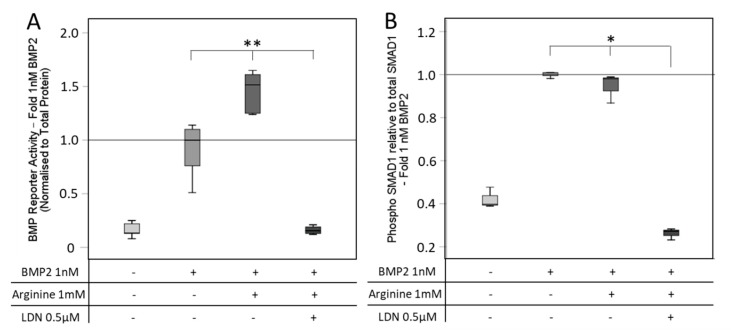
BMP reporter activity and SMAD1 phosphorylation after inhibition of the SMAD pathway. (**A**) C2C12BRELuc cells were stimulated for 6 h in Arginine-free DMEM with 1 nM BMP2 with or without 1 mM Arginine and 0.5 µM LDN. (**B**) C2C12BRELuc cells were stimulated for 30 min in Arginine-free DMEM with 1 nM BMP2 with or without 1 mM Arginine and 0.5 µM LDN. SMAD1 phosphorylation was measured with the SMAD1 InstantOne ELISA™ kit and normalized to total SMAD1. Statistics: Kruskal–Wallis Test followed by Mann-Whitney *U* test. Mann-Whitney *U* test significances between samples were assigned if *p* < 0.05 (*) or *p* < 0.01 (**). Abbreviations: LDN: LDN-193189 (**A**) n = 6; (**B**) n = 3.

The addition of the BMP type-I receptor inhibitor LDN, which inhibits BMP-mediated SMAD signaling, led to a decrease of BMP reporter activity, irrespective of the presence of the NOS substrate L-Arginine (Figure 9A). Analyzing the phosphorylation of SMAD1, this effect could be underlined, as the inhibitor LDN significantly reduced the SMAD1 phosphorylation. Arginine supplementation to BMP2 had no additional effect on the SMAD1 phosphorylation under the tested conditions (Figure 9B).

### 3.5. PKA Connects BMP2 and NO Pathway for BMP2 Signaling

**Figure 10:**
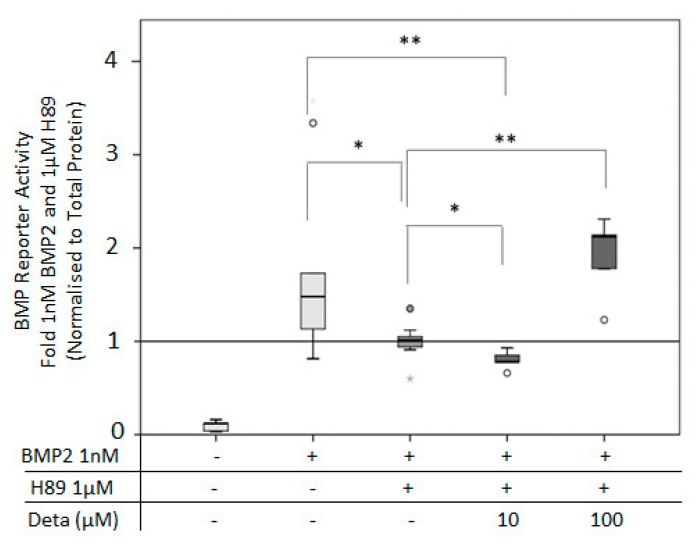
BMP reporter activity after inhibition of protein kinase A (PKA) by H89: BMP reporter activity of C2C12BRELuc cells stimulated for 6 h with 1 nM BMP2, 1 µM H89, 10 and 100 µM Deta NONOate. Statistics: Kruskal–Wallis Test followed by Mann-Whitney *U* test. Mann-Whitney *U* test significances between samples were assigned if *p* < 0.05 (*) or *p* < 0.01 (**). Abbreviation: Deta = Deta NONOate. Circles are outlying values. Replications n = 6.

A PKA-mediated link between the BMP2 and NO pathway has been described [27]. To investigate this connection, the PKA inhibitor H89 was used. Application of 1 µM H89 significantly reduced BMP2-induced signaling. The inhibition of BMP2 signaling was rescued by the application of 100 µM Deta NONOate (Figure 10).

### 3.6. BMP2 Can Induce NO Pathway Activity

**Figure 11:**
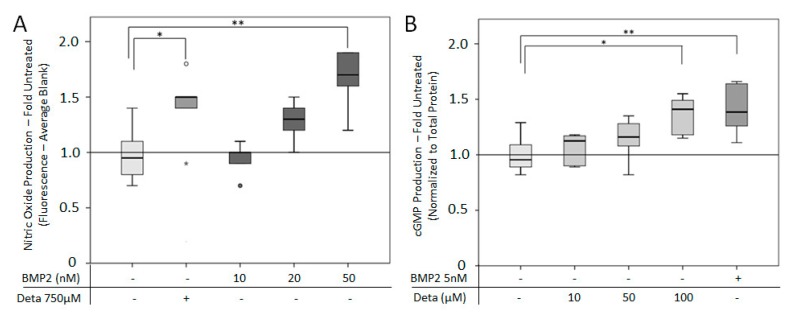
Treating C2C12BRELuc cells with BMP2 stimulated NO signaling. (**A**) NO production quantified by DAF-2-mediated fluorescence and fold comparison to untreated group. C2C12BRELuc cells were stimulated for 2 h with 750 µM Deta NONOate or 10, 20, or 50 nM BMP2. (**B**) cGMP production in cells after 1 h stimulation with 10, 50, or 100 µM Deta NONOate or 5 nM BMP2 given as fold comparison to untreated groups. Statistics: Kruskal–Wallis Test followed by Mann-Whitney *U* test. Mann-Whitney *U* test significances between samples were assigned if *p* < 0.05 (*) or *p* < 0.01 (**). Abbreviation: Deta: = Deta NONOate. Stars and circles indicate outlying values. Replications (A, B) n = 6.

We hypothesized that BMP2 is able to activate the NO pathway. To prove this, NO and cGMP production was examined in response to BMP2 (Figure 11). The quantification of NO was carried out using the fluorescent NO reactive DAF-2. The addition of 10–50 nM BMP2 led to a dose-dependent increase in NO production, but only 50 nM BMP2 was significantly higher compared to the untreated group (Figure 11A) and comparable to the NO donor Deta NONOate. Stimulation of cells with 5 nM BMP2 significantly increased the cGMP production compared to the untreated group. The BMP2-induced cGMP production was comparable to the amount induced by 100 µM Deta NONOate (Figure 11B).

## 4. Discussion

This study aimed to investigate the molecular association between the BMP2 and NO pathways and to gain further understanding of the ability of the NO pathway to enhance BMP2-mediated signaling and osteoinductive activities. To this end, BMP2 was applied in combination with various compounds that stimulate or inhibit the NO pathway in C2C12 cells, and BMP2 reporter activity and osteoinduction (ALP activity) was measured. By demonstrating that the NO pathway can enhance BMP2-mediated signaling and osteoinduction, there are opportunities to enhance BMP2-based therapeutics, e.g., in fracture treatment, in turn filling the current need for increased BMP2 therapeutic efficiency [45].

Utilizing Arginine-free DMEM as a cell culture medium allowed for better control of NO signaling. Therefore, this study clearly demonstrated that NO production by the NOS enzyme can enhance BMP2-mediated signaling. Supplementation of BMP2 stimulation with Arginine enhanced BMP2-mediated reporter activity and the expression of BMP2 target genes *Id1*, *Id2*, and *Id3* after 6 h of stimulation. LNAME inhibition of the NOS enzyme confirmed the NO-BMP2 link. However, high concentrations of LNAMEs were required (5 to 10 mM) to reduce BMP2-mediated signaling. The reason could be the BMP2-mediated activation of the NOS enzyme [27] which, in turn, acts as a competitor to the inhibition by LNAME.

The addition of the direct NO donors SNAP and Deta NONOate for a short period of time (6 h) had no effect on BMP2-mediated signaling. However, SNAP addition without BMP2 was able to induce BMP reporter activity. At a longer period of time (24 h), Deta NONOate enhanced BMP2-mediated signaling, whereas SNAP did not. This provides further evidence that the NO pathway can enhance BMP2-mediated signaling. These time-dependent effects of direct NO donors are well-known due to the variable half-life of the NO donors [46,47,48].

Investigating the phosphorylation of SMAD1, a clear effect of BMP2 was seen and the phosphorylation was reduced by the BMP-receptor inhibitor LDN. As Arginine was not able to recover the inhibition of the BMP2 pathway, NO supplementation might require a functional BMP pathway. The reason for the ineffectiveness of Arginine on the additional stimulation of SMAD1 phosphorylation under BMP2 exposure cannot be explained, although a significant supplementary effect on the reporter activity was detected. Further studies are necessary to elucidate if concentration and time dependency might be responsible.

In this study, the supplementary role of the NO receptor sGC in BMP2-mediated signaling was investigated using the chemical inhibitor LY83583 and the NO independent activator YC-1. Application of the sGC inhibitor LY83583 reduced BMP2-mediated signaling, while addition of YC-1 had a supportive effect on the BMP2-mediated signaling. Taken together, these results demonstrate a role for the NO receptor, sGC, in supporting BMP2-mediated signaling.

To understand if the NO pathway is able to support the early osteogenic differentiation in C2C12 cells, ALP activity and expression of osteogenic markers were investigated. As Arginine-free DMEM did not allow the induction of ALP activity in C2C12 cells, a media containing Arginine was required to investigate BMP2-mediated ALP activity. Therefore, Arginine supplementation could not be used, and the role of NO in BMP2-mediated ALP induction was investigated by the application of direct NO donors and NOS inhibitors. The application of NOS inhibitor (LNAME) in combination with BMP2 inhibited the BMP2-mediated ALP activity, which was partially recovered by the addition of the direct NO donor Deta NONOate. The osteogenic differentiation was further investigated by gene expression analysis of the osteogenic markers *OPG, Runx2*, and *Col1a1*. The analysis revealed that the NO substrate Arginine was able to enhance the osteogenic differentiation compared to BMP2 alone. Altogether, this highlights the role of the NOS enzyme and NO pathway in supporting BMP2-mediated early osteogenic activity, and that the addition of NO donors can help to enhance BMP2-mediated activity.

There are dosing effects between BMP2 and Deta NONOate in the regulation of ALP activity. This work demonstrated that the BMP2-mediated ALP activity of Deta NONOate was only achieved at higher BMP2 concentrations (10 and 20 nM). In addition, while Deta NONOate was supplementary at low concentrations (10 µM), it lost effectiveness and became inhibitory with increasing concentrations. It is interesting to note a parallel effect seen in stretching experiments of C2C12 cells: 10 µM Deta NONOate enhanced stretch induced proliferation, whereas 50 or 100 µM had no effect [49]. Therefore, further studies are needed to determine the NO donor concentration required to achieve the most effective activity, as well as to elucidate the mechanism of adverse effects with increasing NO concentration.

The negative effects of NO on cell vitality were previously described [50,51,52,53]. Particularly, in C2C12 cells, the NO donor SNP reduced the cell number and increased the apoptotic markers p53 and caspase-3 in a concentration- and time-dependent manner [54]. However, in differentiated C2C12 cells treated with 0.35 to 3 mM Deta NONOate for 24 h, viability studies found that at the lowest concentration (0.35 mM), cell viability was 82.1%, while at the highest concentration (3 mM), cell viability dropped to 41.5% [55]. Over 24 h Deta NONOate was able to enhance stretch-induced proliferation of C2C12 at 10 µM, but this was lost at higher concentrations (50–100 µM) and did not reduce proliferation compared to control [49]. In the C2C12BRELuc cells used in this study, the application of 10 to 100 µM Deta NONOate had little to no negative effects on C2C12BRELuc viability over 24 h. A decrease in p53, however, might also be associated with osteogenic differentiation as reported earlier for mouse bone marrow-derived mesenchymal cells [56]. The authors found that p53 indirectly suppresses *Runx2* expression.

In clinical use, a high concentration of BMP2 is applied, possibly contributing to the negative side effects seen in patients [57,58]. The ability of Arginine, YC-1, and Deta NONOate to supplement BMP2-mediated signaling and early osteogenic differentiation provides an opportunity to reduce the amount of BMP2 applied in the clinic while maintaining the osteoinductive effects. As NO pathway activators (such as nitroglycerine) have been previously used in clinical trials as an orthopedic therapy [34,35,59,60], this should provide a valuable starting point for future investigations into compounds that can be applied clinically in combination with BMP2.

Furthermore, it was noticed that Deta NONOate-, SNAP-, and YC-1-mediated activation of the NO pathway led to a BMP2-independent activation of BMP reporter activity. This could be an indication of an increase in *BMP2* expression, as shown in cultured embryonic heart cells in response to NO pathway activation [61], or though osteoinduction as seen in MC3T3 cells [62,63].

It was previously shown that PKA connects BMP2 signaling with the activation of eNOS through phosphorylation in pulmonary cells [27]. This possible link was investigated now in a more osteogenic context. The application of the PKA inhibitor H89 led to inhibition of BMP2-mediated signaling, which was recovered by the addition of the direct NO donor, Deta NONOate. This underlines a connection between BMP2, PKA, and the NO pathway. This finding is in agreement with results reported in previous work, where it was shown that PKA activation itself leads to osteoinduction in MC3T3 cells [64,65], also involving the PKA/CREB signaling [66].

This study showed the ability of the BMP2 pathway to activate the NO pathway through an increase of NO and cGMP production in C2C12 cells. BMP2-stimulated NO production was clearly dose-dependent, with NO production increasing with higher BMP2 concentrations. This is in line with previous studies, where increasing BMP2 concentration increased the NO production in chondrocytes [67]. In endothelial cells, BMP2 enhanced the NOS activity. However, the BMP2 dosage effects on NOS activity was not investigated [27]. This study required a high concentration (50 nM) of BMP2 to significantly increase NO production compared to other studies [27,67], which was partially due to the short stimulatory time required for DAF-2-mediated NO quantification (2 h) compared to the longer times point of Griess assay (4 d) used for BMP2 treated chondrocytes [67]. The BMP2-mediated activation of the NO pathway also increased the cGMP level. This builds on previous studies, which have focused on the association of the BMP2 signaling with the cGMP-activated cGKI. It was demonstrated that the addition of 8-br-cGMP increased cGKI binding of the BMPR2 tail domain [26], but previous studies have overlooked whether addition of BMP2 leads to an increase in cGMP production. Collectively, this and previous work [26,27,67] suggest a possible positive reinforcement of BMP2 signaling, whereby BMP2 is enhanced by the NO pathway and BMP2 itself can activate the NO pathway.

This work provided evidence that the NO pathway is capable of enhancing BMP2-mediated signaling and early osteogenic differentiation, which can have implications for the application of BMP2 in the clinic. Further, it was demonstrated that PKA connects BMP2 and NO signaling. This investigation has solidified the role of NO in activating the BMP2 pathway by demonstrating this in the C2C12 cells. Further studies should investigate the effect on osteogenic differentiation in vitro and in vivo by addressing the effect on mineralization in vitro and ectopic, as well as heterotopic bone formation in vivo.

## 5. Conclusions

**Figure 12:**
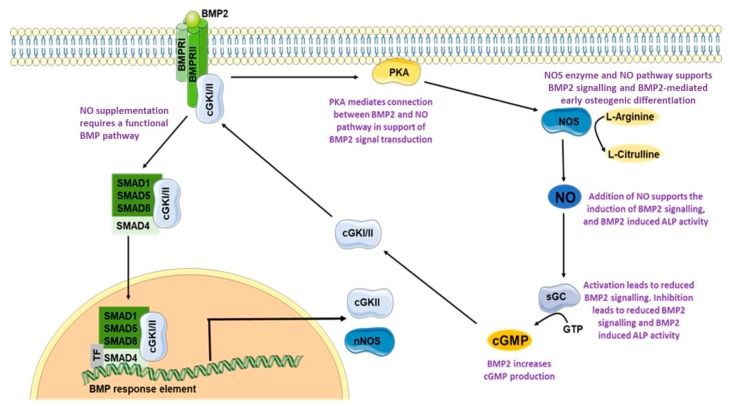
Graphical summary of perceived crosstalk of BMP2-mediated signaling and the NO pathway. Figure image composed from graphics taken from Servier Medical Art.

The presented data show that there is a crosstalk between the NO and BMP signaling pathway and that NO leads to enhanced BMP activity (summarized in Figure 12). The NO pathway is linked to the BMP pathway via sGC, with PKA mediating the link between BMP2 and NO pathway.

## Figures and Tables

**Table 1:** Experimental setups for this study.

Method	Pre-Stimulation		Stimulation		
	Media	Additional Treatment	Media & FCS	Added Factors	Time (h)
Reporter activity	DMEM without Arginine, 0.1% FCS	Inhibitors: LNAME, LY83583, H89, LDN final hour	DMEM without Arginine, 0.1% FCS	Arginine, Deta, SNAP and BMP2	24 6
ALP activity	DMEM/HAMs, 1% FCS	Inhibitors: LNAME, LY83583 final hour	DMEM/HAMs, 1% FCS	Arginine, Deta, and BMP2	72
NO production	DMEM High Glucose, 1% FCS	DAF-2, 1 h	DMEM High Glucose, 1% FCS	Deta, and BMP2	2
cGMP ELISA	DMEM without Arginine, 1% FCS	IBMX final 30 min	DMEM without Arginine, 1% FCS	Deta, and BMP2	1
SMAD-1 ELISA	DMEM without Arginine, 0.1% FCS	Inhibitors: LDN, final hour	DMEM without Arginine, 0.1% FCS	Arginine and BMP2	0.5
Gene Expression ***Id1,2,3***	DMEM without Arginine, 0.1% FCS	-	DMEM without Arginine, 0.1% FCS	Arginine, and BMP2	6
Differentiation (Gene Expression ***OPG, Runx, Col1a1***)	DMEM without Arginine, 1% FCS	-	DMEM without Arginine, 1% FCS	Arginine and BMP2	144

**Table 2:** Murine primers for quantitative real-time polymerase chain reaction (qRT-PCR).

Gene	Accession Number	Forward (5′→3′)	Reverse (5′→3′)
*HPRT*	NM_000194	TTGCTGACCTGCTGGATTAC	AACTTTTATGTCCCCCGTTG
*Id1*	NM_010495.3	ACGACATGAACGGCTGCTACT	GCTCACTTTGCGGTTCTGG
*Id2*	NM_010496.3	TATCAGCCATTTCACCAGGAG	TGTGAAAAGGCAAAGTCTGCT
*Id3*	NM_008321	AAGGACAAGAGGAGCTTTTGC	GCGTTGAGTTCAGGGTAAGTG
*OPG*	NM_002546.3	TGCAGTACGTCAAGCAGGAG	CCCATCTGGACATCTTTTGC
*Col1a1*	NM_000088.3	TGACCTCAAGATGTGCCACT	ACCAGACATGCCTCTTGTCC
*Runx2*	NR_103532	GCCCCCAAACAGTATCTTGA	GCCTGAAGTGAGGTTTTAGGC

## Supplementary Materials

The following are available online at https://www.mdpi.com/2073-4409/8/10/1273/s1, Figure S1: Supplementary figure for melting curves of qRT-PCR.

## Abbreviations

*18S*	ribosomal unit 18S	LDN	LDN-193189
ALP	Alkaline Phosphatase	Luc	Luciferase
AMHR	anti-Mullerian hormone receptor type	MNE	mean normalized expression
Arginine BMP	L-Arginine Bone Morphogenic Protein	nNOS	neuronal Nitric Oxide Synthase
BMPR	BMP receptor	NO *OPG*	Nitric Oxide Osteoprotegerin
BMPR2-LF/-SF	BMP receptor - long form/- short form	PAEC	pulmonary arterial endothelial cells
BRE	BMP response elements	PBS	Phosphate Buffered Saline
cGKI/II	cGMP dependent kinase I/II	PDE	phosphodiesterase’s
cGMP	cyclic guanosine monophosphate	Pen/Strep	Penicillin-Streptomycin
CO_2_ *Col1a1*	Carbon Dioxide Type 1 collagen	PKA	Protein Kinase A
Co-SMAD	common mediator SMAD	pNPP	4-nitrophenyl phosphate
CREB	cAMP response element-binding -	PP2	protein phosphatase 2
CT	Crossing Threshold	rhBMP2	recombinant human BMP2
DAF-2T	4,5-Diaminofluorescein triazole	RLU	relative light units
Deta	Deta NONOate	R-SMAD	receptor regulated SMAD
DMEM	Dulbecco Modified Eagle Medium	*Runx2*	Runt-related transcription factor 2
ECM	extracellular matrix	sGC	soluble Guanylyl Cyclase
ELISA	Enzyme Linked Immunosorbent Assay	SMAD	Small Mothers Against Decapentaplegic
eNOS	Endothelial Nitric Oxide Synthase	TF	Transcription Factors
Exp.	Experiment	TGFβ	Transforming Growth Factor β
FCS	Fetal Calf Serum		
*Id1,2,3*	*Inhibitor of Differentiation 1,2,3*		
