# Supplementary material for: Is NO the Answer? The Nitric Oxide Pathway Can Support Bone Morphogenetic Protein 2 Mediated Signaling
Source: Cells. 2019 Oct 18;8(10):1273. doi: 10.3390/cells8101273 (PMC6830101; doi:10.3390/cells8101273)
Supplement: Supplementary file 1 [file cells-08-01273-s001.pdf]

Supplementary figure for melting curves of qRT-PCR:

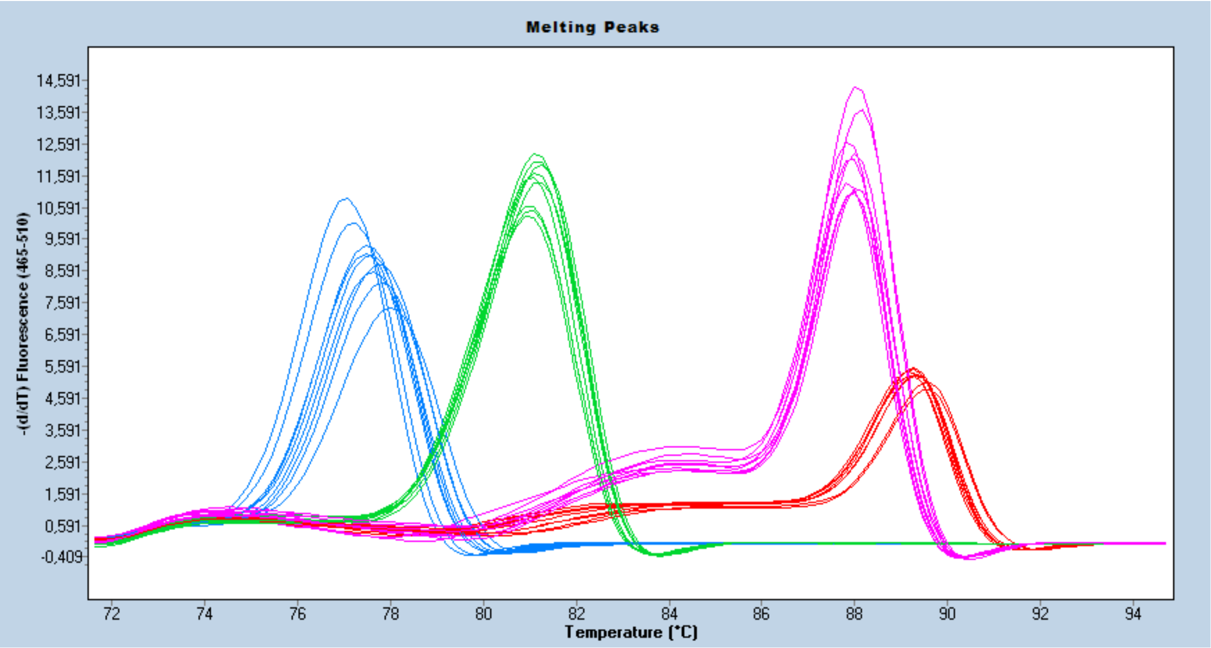

Figure S1: Melting curves for *HPRT* (blue), *Id1* (red), *Id2* (green), *Id3* (pink) for unstimulated C2C12 cells and cells stimulated with BMP2 (1nM) with and without arginine.
